# Human gut microbial communities dictate efficacy of anti-PD-1 therapy in a humanized microbiome mouse model of glioma

**DOI:** 10.1093/noajnl/vdab023

**Published:** 2021-02-08

**Authors:** Kory J Dees, Hyunmin Koo, J Fraser Humphreys, Joseph A Hakim, David K Crossman, Michael R Crowley, L Burton Nabors, Etty N Benveniste, Casey D Morrow, Braden C McFarland

**Affiliations:** 1 Department of Cell, Developmental and Integrative Biology, University of Alabama at Birmingham, Birmingham, Alabama, USA; 2 Department of Genetics, University of Alabama at Birmingham, Birmingham, Alabama, USA; 3 Department of Neurology, University of Alabama at Birmingham, Birmingham, Alabama, USA; 4 School of Medicine, University of Alabama at Birmingham, Birmingham, Alabama, USA

**Keywords:** anti-PD-1, glioblastoma, GL261, immunotherapy, microbiome

## Abstract

**Background:**

Although immunotherapy works well in glioblastoma (GBM) preclinical mouse models, the therapy has not demonstrated efficacy in humans. To address this anomaly, we developed a novel humanized microbiome (HuM) model to study the response to immunotherapy in a preclinical mouse model of GBM.

**Methods:**

We used 5 healthy human donors for fecal transplantation of gnotobiotic mice. After the transplanted microbiomes stabilized, the mice were bred to generate 5 independent humanized mouse lines (HuM1-HuM5).

**Results:**

Analysis of shotgun metagenomic sequencing data from fecal samples revealed a unique microbiome with significant differences in diversity and microbial composition among HuM1-HuM5 lines. All HuM mouse lines were susceptible to GBM transplantation, and exhibited similar median survival ranging from 19 to 26 days. Interestingly, we found that HuM lines responded differently to the immune checkpoint inhibitor anti-PD-1. Specifically, we demonstrate that HuM1, HuM4, and HuM5 mice are nonresponders to anti-PD-1, while HuM2 and HuM3 mice are responsive to anti-PD-1 and displayed significantly increased survival compared to isotype controls. Bray-Curtis cluster analysis of the 5 HuM gut microbial communities revealed that responders HuM2 and HuM3 were closely related, and detailed taxonomic comparison analysis revealed that *Bacteroides cellulosilyticus* was commonly found in HuM2 and HuM3 with high abundances.

**Conclusions:**

The results of our study establish the utility of humanized microbiome mice as avatars to delineate features of the host interaction with gut microbial communities needed for effective immunotherapy against GBM.

**Key Point**

- We have created a unique model in which mice are colonized by human microbial communities in the GI tract, which allows us to investigate the role of human microbial communities on the growth and response to therapies in a preclinical mouse model of glioma.

**Importance of the Study**

Although immunotherapy works well in GBM preclinical mouse models, the therapy has not demonstrated efficacy in GBM patients. While the reason for this discrepancy is unknown, all GBM preclinical studies to date have been done in mouse models using mouse gut microbiomes. We hypothesize that the gut microbiome may influence the response of GBM patients to immunotherapy. Therefore, we employed a unique model in which mice are colonized with human microbial communities from 5 different healthy donors to generate 5 unique humanized microbiome mouse lines to test the growth and response to GBM growth in mice. We found that the human microbial communities in the GI tract of the mice influenced the response to immunotherapy, with some exhibiting a beneficial response and others being nonresponsive. This is the first study to examine how human microbial communities influence the growth and response to therapies in a preclinical model of GBM.

Patients diagnosed with glioblastoma (GBM), the most common primary malignant brain tumor, have a grim prognosis and a median survival of only 12–16 months despite aggressive treatment regimens.^1^ Recently, the field of immunotherapy (promoting an anti-tumor immune response) has demonstrated successful and exciting results in melanoma and lung cancer.^2^ These efforts include antibodies that block the immunosuppressive signals on T-cells to activate an anti-tumor response, termed immune checkpoint inhibitors (ICI), and include anti-programmed death ligand 1 (PD-1) and anti-cytotoxic T-lymphocyte-associated protein 4 (CTLA-4). Studies have shown that successful ICI treatment is associated with increased cytotoxic CD8^+^ (granzyme-B^+^ or IFN-γ ^+^) T-cells and reduced Tregs in the tumor.^3,4^ The first large clinical trial of ICI testing anti-PD-1 in recurrent GBM (CheckMate-143) found that there was a failure of anti-PD-1 (with or without combination anti-CTLA-4) to prolong overall survival compared to the control of bevacizumab.^5,6^ However, 3 studies found that that there is a positive T-cell response following anti-PD-1 treatment in GBM patients, and that altering the dosing schedule may provide better response.^7–9^

There is a growing awareness for the role of the gut microbiome in the development and function of the immune system.^10,11^ Furthermore, there is extensive crosstalk and interaction between the gut microbiota and the brain, termed the gut-brain axis.^12^ Many of these interactions have been studied through the use of gnotobiotic (germ-free) mice, which have no live microbes in the GI tract. These animals have defective immune responses that can be restored by microbe transplant. Surprisingly, the composition of the gut microbiome has been shown to promote resistance to immunotherapy in melanoma and other cancers.^13–15^ In 2015, it was reported that mice with different gut microbiomes exhibited different rates of tumor growth, and immunotherapy was ineffective in gnotobiotic mice or mice that have been treated with antibiotics.^14,15^ In 2018, reports evaluated primarily melanoma patient gut microbiomes, including metastatic melanoma patients, and identified responders and non-responders to immunotherapy.^13^ More recently, a study found that the community of commensal microbes per individual, not just one species of bacteria, act as a consortium to produce a maximal immune response and effectiveness of immunotherapy in tumor models.^16^ However, to date, no studies have evaluated the role of the gut microbiome and response to immunotherapy in GBM.

Although anti-PD-1 works well in GBM preclinical mouse models, the therapy has not demonstrated a similar efficacy in patient clinical trials.^5,7,8,17^ We note, that to date, all GBM pre-clinical studies have been done in mouse models using mouse gut microbiomes. There are significant differences between mouse and human microbial gut compositions, and some studies have found that 85% of gut bacteria found in laboratory mice are not found in humans.^18^ Therefore, we have utilized a humanized microbiome (HuM) model, in which mice have been colonized by healthy human donor microbial communities, to determine the effect of the various microbial communities and response to therapy in a preclinical mouse model of GBM. In this study, we examined the growth of GL261 murine brain tumors and response to ICI therapy in HuM mice, which are genetically identical and only differ in the composition of the gut microbiome. Interestingly, we have found that the HuM microbiome composition of the mice influences the efficacy of anti-PD-1 therapy in mice with intracranial tumors, with some HuM mice exhibiting resistance and other HuM mice displaying a positive anti-tumor response to anti-PD-1. Overall, this study indicates a prominent and important role of the gut microbiome in the response to immunotherapy in a GBM preclinical model.

## Materials and Methods

### Ethics Statement

All experiments with mice (male and female) were performed with the approval of the University of Alabama at Birmingham (UAB) Institutional Animal Care and Use Committee (#IACUC-21220, IACUC-21645, and IACUC-21922). Consent form for human fecal samples was obtained as part of an ongoing IRB-approved study at UAB IRB300004198. Informed consent from all donors was obtained.

### Cells and Reagents

GL261 cells were a kind gift from Dr. G. Yancey Gillespie (University of Alabama at Birmingham). Cells were grown at 37°C and 5% CO_2_ in DMEM/F12 media supplemented with 10% FBS and penicillin/streptomycin and L-glutamine as described.^19^ Immunotherapy antibodies anti-PD-1 (InVivoMAb clone #RMP1-14) and anti-isotype control (InVivoMab rat IgG2a isotype control clone #2A3) were purchased from BioXcell. Temozolomide was purchased from Cayman chemicals. Oraplus solution was purchased from Amazon.

### Generation of HuM Mice

The generation and validation of the humanized microbiome mouse model are detailed as previously described.^20^ Briefly, fecal samples were collected with informed consent from healthy human donors. Samples were cryogenically preserved to allow fecal transplantation into gnotobiotic mice. Gnotobiotic mice used that were available from the UAB Gnotobiotic core are 10BitFoxP3.GFP B6 mice, which are BL/6 background mice and appropriate for the GL261 model of glioma.^21^ Gnotobiotic mice were given 100–200 μL of donor fecal material via oral gavage to establish humanized microbiome mice as described.^20^ Mice were bred and the progeny of these lines were used for all experiments herein. For control mice, gnotobiotic mice of the same genetic strain (10BitFoxP3.GFP B6) were given a transplant of a fecal sample from a WT C57BL/6 mouse to mimic a murine microbiome (MuM) mouse and bred for experiments.

### Isolation of Microbial DNA and Metagenomic Sequencing

Fecal samples were collected from the HuM mice, microbial DNA was isolated using the Zymo Fecal DNA Isolation kit, and shotgun metagenomic sequencing was performed as described.^20^ The metagenomics sequencing FASTQ files used in this study were previously deposited in the National Center for Biotechnology Information BioProject under accession number PRJNA593263.^20^

### Taxonomic Profiles of the HuM Mice and Diversity Analyses

Taxonomic profiles for healthy human donor fecal samples, breeders, and their F1 progenies (mice born from the humanized microbiome breeder mice) were analyzed as previously described.^20^ In this study, we have selected the taxonomic profiles from only F1 progenies to further conduct diversity analyses. The taxa (species level resolution) abundance table which included “estimated number of reads from the clade” values was standardized and then used to determine the Bray-Curtis distance matrix values using vegan R package.^22,23^ A cluster dendrogram was created based on the Bray-Curtis distance matrix using “hclust (method = average)” function in the vegan R package.^22,24^ Nonmetric multidimensional scaling (NMDS) plot was also generated to show variation between each sample using vegan R package.^22^ In the NMDS plot, samples were grouped by ellipses with a confidence interval of 95% using vegan R package^22,25^ Shannon diversity measurements were determined using vegan R package^22^ and plotted for all humanized mice. The Venn diagram plot was created to represent the shared and unique microbial species among the 5 HuM groups using the gplots^26^ and the VennDiagram^27^ packages in R.

### Intracranial Injections

HuM mice (HuM1-HuM5) or MuM mice ages 6–12 weeks were used for the intracranial tumor experiments, and tumor injections performed as previously described.^19^ For the immunotherapy experiments, mice were randomized to receive immunotherapy (anti-PD-1 or isotype control) starting on day 5 postintracranial injection. Mice were given anti-PD-1 (200 μg) or isotype control (200 μg) via i.p. injection once every 3 days for a total of 4 doses. Mice were monitored for survival and euthanized upon neurological signs of tumor burden. Mice that unexpectedly died prior to the end of treatment and without visible tumor growth were excluded. For the TMZ experiments, mice were randomized on day 5 post-tumor injection to receive TMZ (25 mg/kg) or vehicle (Oraplus) via oral gavage twice a week for 2 weeks. Mice were monitored for survival and euthanized upon moribund. Brains were formalin fixed, paraffin embedded and sectioned (8 μm) for H&E staining by the Comparative Pathology Core (UAB).

### Statistics

For all microbiome-related sample analysis, metagenomics sequencing and analyses was performed by the UAB Microbiome Resource. For the Shannon diversity box plot, statistical significance (*P* value < .05) was determined by using 1-way ANOVA followed by Tukey’s multiple-comparisons post-hoc test in R software.^28^ Differences in microbial community structure between groups were measured using permutational multivariate analysis of variance (PERMANOVA) with the function ADONIS in the vegan R package.^22^ For survival analysis, LogRank was used to determine statistical significance between survival curves. For all other analyses, Student’s *t*-test was used to compare 2 samples and a 1-way ANOVA with Tukey’s post-hoc test was used to compare 3 or more samples. If normal distribution failed, then Mann–Whitney test was used for two comparisons and Kruskal–Wallis test used for more than two comparisons. For all statistical analyses, a *P* value of <.05 was considered statistically significant.

## Results

### Generation of Humanized Microbiome Mice

We transferred 5 healthy human donor fecal samples (via oral gavage) into gnotobiotic mice to establish humanized microbiome (HuM) mice: HuM1, HuM2, HuM3, HuM4, and HuM5. We then established breeder pairs of these mice by housing with gnotobiotic male mice, which maintains the human microbes in the GI tract and creates a novel colony model system. We have recently published the method of our HuM model and confirmed that specific microbial communities (at the species level) are successfully passed on to the progeny.^20^ For this study, F1 progenies from each of the HuM lines were used for the experiments and analyses herein.

### Significant Differences in Microbial Communities, Diversity, and Clustering Between the Humanized Mice

**Figure 1.**
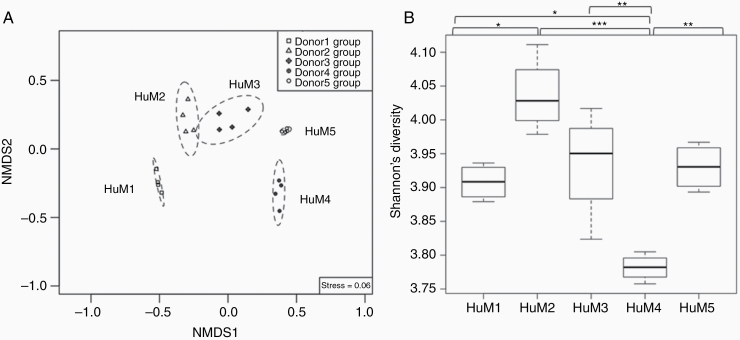
Significant differences in microbial communities, diversity and clustering between the humanized mice. (A) Nonmetric multidimensional scaling (NMDS) plot shows overall differences in the microbial community structure at the species level across all humanized mice (*n* = 4 per group; *n* = 20 total). A significant difference (*R*^2^ = 0.94, *P* value = 1e-04) in microbial community structure across all groups was supported by using permutational multivariate analysis of variance (PERMANOVA) with the function ADONIS in the vegan R package. (B) Shannon diversity was measured and plotted for all humanized mice using vegan R package. The line inside the box represents the median, while the whiskers indicate the lowest and highest values within the 1.5 interquartile range (IQR). Significant differences (*P* value <.05) between each group were tested using an ANOVA followed by Tukey’s multiple-comparisons post-hoc tests in R (version 3.5.1), and represented as a black asterisk above the boxplot; *P value <.05, **P value <.01, ***P value <.001, not significant values are not shown in the boxplot.

Fecal samples from the HuM mice (HuM1-HuM5) were collected and analyzed for metagenomic sequencing in order to confirm differences in gut microbiota between each group. We found significant differences in the microbial community structure between HuM1-HuM5 as shown by distinct clustering in the NMDS plot (Figure 1A). When comparing diversity within a single HuM group, Shannon diversity analysis found that HuM2 exhibited the highest average value compared to the other HuM mice and was significantly higher than HuM1 and HuM4 (Figure 1B). HuM3 mice displayed the second highest average value and was significantly higher than HuM4 mice. HuM4 microbiome mice had the lowest value compared to all other HuM mice. Overall, this confirms that each of the 5 HuM mice lines are distinct and significantly different from one another.

### Analysis of Intracranial Glioma Growth in Humanized Microbiome Mice

**Figure 2.**
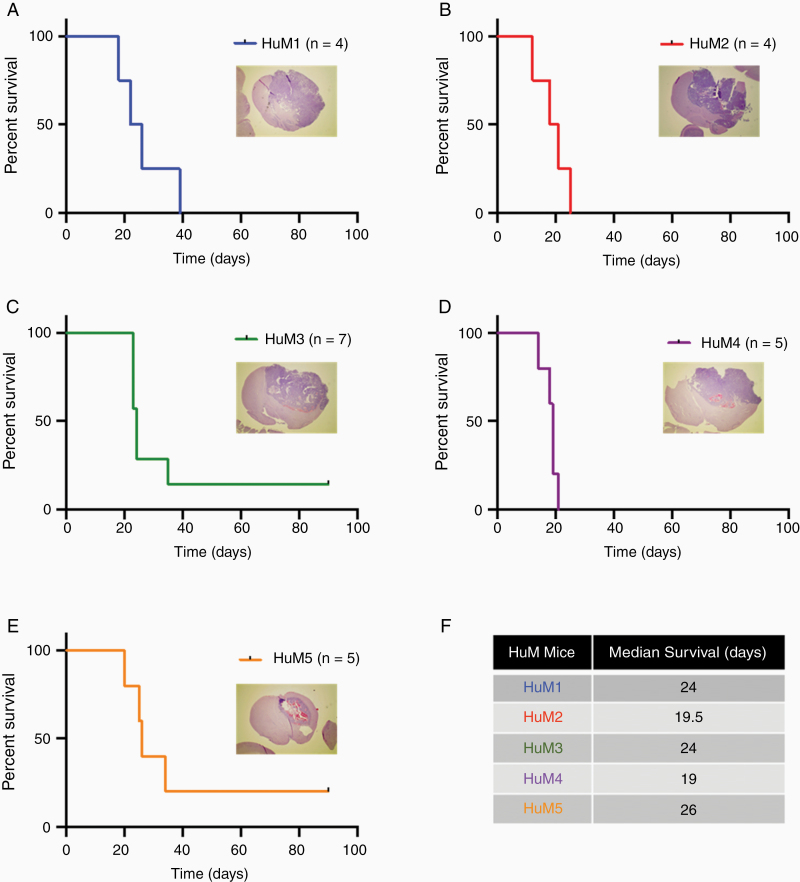
Intracranial tumor growth and survival is comparable among HuM mice. HuM mice were injected with GL261 cells (1 × 10^5^ cells/5 μL). Mice were monitored for survival and euthanized at moribund. (A) HuM1 *n* = 4 females; (B) HuM2 *n* = 4 females; (C) HuM3 *n* = 7 females and males; (D) HuM4 *n* = 5 females; and (E) HuM5 *n* = 5 females. Insets, H&E microscopy images were taken at 12.5×. (F) Median survival for each of the 5 HuM groups. Experiments were performed at different times and were presented as individual curves for clarity.

Next, we wanted to examine how the different microbiome communities would affect tumor growth in the HuM mice using the GL261 syngeneic preclinical model of glioma. No previous reports have analyzed glioma growth in mice with human microbes colonizing the GI tract. We injected HuM mice (HuM1 – HuM5) with GL261 glioma cells to determine if human microbes in the GI tract affect tumor growth and overall survival. We found that glioma tumors grew in all 5 groups of HuM mice, and the overall survival curves are comparable between groups (Figure 2A–E). To confirm tumor growth in these mice, a representative H&E image of a mouse brain with prominent tumor growth for each HuM mouse is shown (Figure 2A–E, inset). The tumors are highly angiogenic, necrotic, and invasive as demonstrated by the histology analysis for each of the HuM mice. Additionally, the median survival times of the HuM mice are shown and vary between the shortest at 19 days (HuM4) to the longest at 26 days (HuM5) (Figure 2F). Overall, this indicates that humanized microbe communities do not overtly impact or prevent the growth of intracranial glioma tumors in mice.

### Humanized Microbiome Mice Exhibit Different Responses to Anti-PD-1 in a Glioma Model

**Figure 3.**
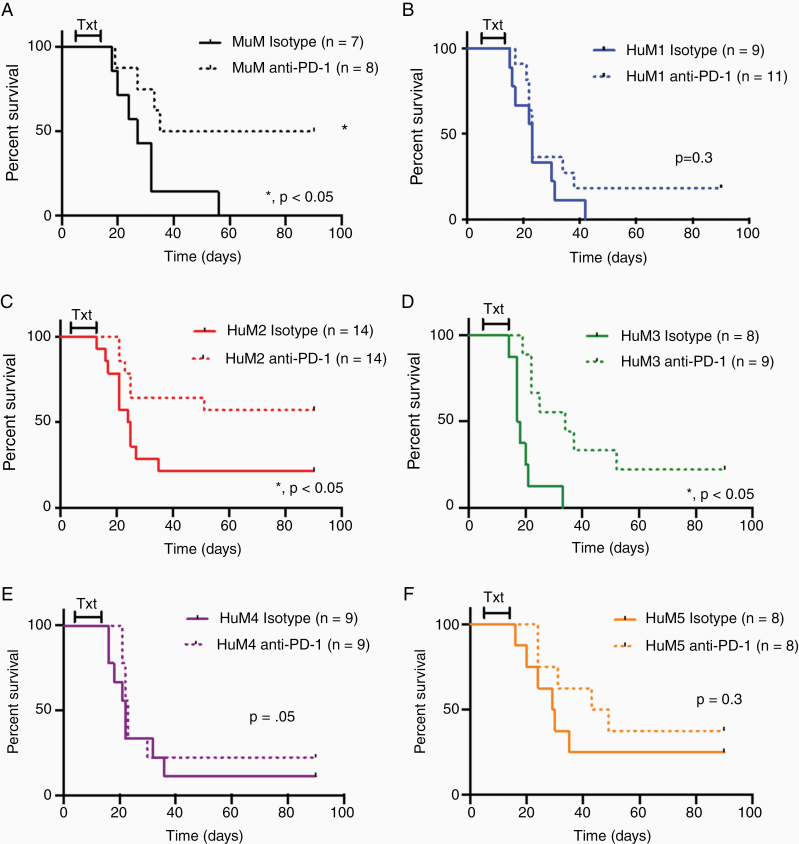
Anti-PD-1 immunotherapy efficacy varies with microbiome differences. (A–F) Murine microbiome (MuM) mice (A) and Humanized microbiome (HuM) mice (HuM1-HuM5; B–F) were intracranially injected with GL261 cells (1 × 10^5^ cells/5 µL). Mice were randomized on day 5 postinjection to receive anti-PD-1 (200 µg) or Isotype Control (200 µg) via i.p. injection every 3 days for a total of 4 doses. Mice were monitored for survival and euthanized at moribund. Male and female mice were used in all experiments. One experiment (E), combination of 2 experiments (A, B, D, F) or 3 experiments (C) are shown for the curves. *, LogRank analysis *P* < .05.

As others have reported the microbiome can influence response to immunotherapies, we examined the ability of anti-PD-1 to prolong survival in our HuM mice with brain tumors. MuM (murine microbiome as controls) mice and HuM1-HuM5 (humanized microbiome) mice were intracranially injected with GL261 cells and on day 5, mice were randomized to receive anti-PD-1 or isotype control. As expected and reported by others,^17^ anti-PD-1 significantly prolonged survival of MuM mice with intracranial tumors (Figure 3A). When examining the response to anti-PD-1 in the HuM mice, we found that anti-PD-1 also significantly prolonged survival in the HuM2 and HuM3 mice (Figure 3C and D). This indicates that HuM2 and HuM3 are responders to anti-PD-1. Surprisingly, anti-PD-1 was unable to prolong survival in the HuM1, HuM4, or HuM5 mice (Figure 3B,E,F), indicating resistance to anti-PD-1. This inability of anti-PD-1 to prolong survival in HuM1, HuM4, and HuM5 mice mirrors the resistance observed in GBM patients. Overall, this indicates that human microbiota can influence the response to immunotherapy, and that some HuM mice are resistant (HuM1, HuM4, and HuM5) to anti-PD-1 therapy, while other HuM mice are responders (HuM2 and HuM3).

### HuM2 Mice Display an Enhanced CD4^+^ and CD8^+^ T-cell Response Following Anti-PD-1 Treatment

Others have reported that baseline T-cell percentages and cytokine expression can predict response to immunotherapy.^16,29^ Therefore, we next examined the peripheral T-cell response to anti-PD-1 in naive mice (nontumor) for wild-type (WT; C57BL/6), a resistant line (HuM1) and a responder line (HuM2). Mice were randomized to receive anti-PD-1 or isotype control via i.p. injection every 3 days for a total of 4 doses to mimic the tumor experiment dosing regimen. We found that HuM2 mice exhibited a significant increase in cytotoxic CD8^+^ and CD4^+^ T-cells producing IFN-γ (Supplementary Figure 1A and B) following anti-PD-1 treatment, which was not observed in the HuM1 mice. We did not detect a significant difference in the percentage of Tregs in mice treated with anti-PD-1 (Supplementary Figure 1C), but a significantly increased CD8^+^/Treg ratio (Supplementary Figure 1D) was revealed in the HuM2 mice following anti-PD-1 treatment. Others have reported that the ratio of CD8/Tregs can be used as a marker for successful anti-tumor response to immunotherapy.^17^ Most importantly, HuM1 CD8^+^ T-cells did not display an increase following anti-PD-1, indicating possible CD8^+^ T-cell dysfunction or anergy in HuM1 mice (Supplementary Figure 1A).

### Standard of Care Temozolomide Maintains Efficacy in Humanized Microbiome Mice

We also examined if temozolomide (TMZ), the standard of care chemotherapy for patients with GBM, is still efficacious in mice with humanized microbiomes. Similar to the immunotherapy experiments, MuM mice and HuM mice (HuM1-HuM5) were intracranially injected with GL261 cells and were randomized to receive TMZ or vehicle control. As expected, TMZ significantly prolonged survival in the MuM mice (Supplementary Figure 2A). Interestingly, we found that TMZ treatment also prolonged survival in the HuM mice (HuM1-HuM4; Supplementary Figure 2B–E). This indicates that the various microbial communities do not have a negative effect on the ability of TMZ to exert anti-tumor effects. However, HuM5 mice did not exhibit a significant prolonged survival, due to one long-term vehicle survivor, but did exhibit the same trend as the others (Supplementary Figure 2F). Overall, TMZ efficacy does not appear to waiver in effectiveness in mice with different human microbiomes.

### Taxonomic Distribution of Microbial Communities in the HuM Mice

**Figure 4.**
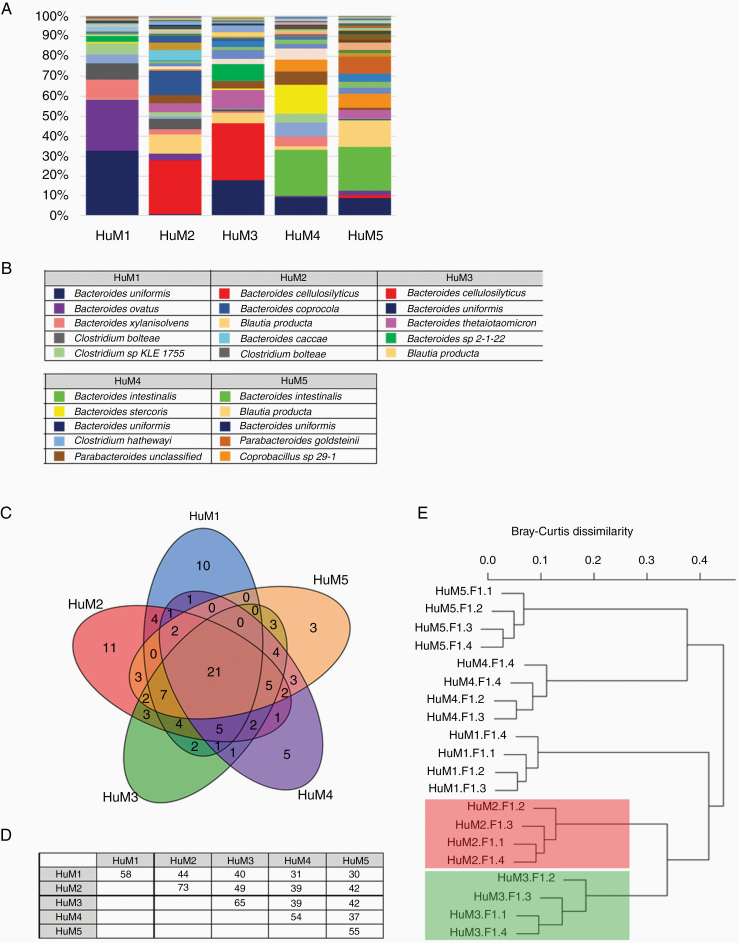
Taxonomic distribution of microbial communities in the HuM mice. (A) The relative abundance of 111 observed species across all HuM mice are shown. Averages for each HuM strain are shown (*n* = 4 mice per group). (B) The top 5 species are listed for each of the HuM mice. (C) A Venn diagram representing shared and unique microbial species among the 5 HuM groups. (D) Pairwise comparison matrix for the 5 HuM groups. Additional shared microbes between 2 or more HuM groups are shown in Supplementary Table 3. (E) Bray-Curtis dendrogram analysis represents significant and distinct clusters specific to each group. HuM2 and HuM3 cluster groups are highlighted in red and green, respectively.

We found that colonization of mice with human microbial communities influences the therapeutic response of anti-PD-1 for mice with intracranial tumors. Specifically, HuM2 and HuM3 mice are responsive to anti-PD-1 and display prolonged survival, whereas HuM1, HuM4, and HuM5 are resistant to anti-PD-1. Therefore, we next examined more closely the taxonomic distribution and relative abundance of microbes at the species level in the HuM mice in order to assess potential unique microbial species that may be contributing to the observed phenotypes. The relative abundance of the total species detected during our analyses is shown as an average of sampled progeny for each group (Figure 4A), and the top 5 species with regards to relative abundance are listed for each HuM mouse line (Figure 4B). The total taxonomic distribution data for the averaged HuM groups is shown in Supplementary Table 1, and total taxonomic distribution data for each individual HuM sample is shown in Supplementary Table 2.

We found that both HuM2 and HuM3 mice exhibited high levels of *Bacteroides cellulosilyticus.* Additionally, HuM2, HuM3, and HuM5 mice had high levels of *Blautia producta*. HuM1 mice appeared to be the only group exhibiting an abundance of *Bacteroides ovatus* in the top 5 relative abundant microbes. HuM4 and HuM5 mice exhibited a high relative abundance of *Bacteroides intestinalis* and *Bacteroides uniformis.* High levels of *B. uniformis* were found in the top 5 for almost all groups, except HuM2. Overall, these data illustrate and confirm that HuM mice display varying abundances of human microbes at the species level.

### HuM Mice Have Shared and Unique Microbial Species

Rather than focusing on one particular microbial species in the HuM lines, we next examined how many and which microbes are shared or unique among the HuM mice. Each of the HuM mice has a unique microbiome composition, but there are species that are both shared and unique to each line. The Venn diagram illustrates the complex overlap between shared and common species for each of the HuM groups (Figure 4C), and a pairwise comparison matrix is shown for the 5 HuM groups (Figure 4D). The values shown in the table represents the number of common species found in each selected pairwise comparison (ie, HuM1 vs HuM1 = 58 total species detected; HuM1 vs HuM2 = 44 total species shared). In looking at HuM2 and HuM3 responder mice, we found that together these mice share 3 unique microbes that are not shared among the other HuM mice, which include *Alistipes indistinctus*, *Blautia hydrogenotrophica*, and *Eubacterium limosum,* but are all in relatively low abundance (Supplementary Table 3). We also found that there are unique microbes that are exclusively found in each HuM mouse line, which are listed in Table 1. For example, HuM2 mice have 11 microbes that are unique to only HuM2. Specifically, *Bacteroides coprocola* and *Bacteroides caccae* are 2 microbes that are only found in HuM2 mice, and both are in the top 5 abundance for HuM2. HuM3 mice have 5 microbes that are unique to only HuM3 (Table 1), but all are in very low abundance.

**Table 1.** Taxa Unique to Each HuM Line

HuM1	HuM2	HuM3
*Bacteroides_massiliensis*	*Alistipes_senegalensis*	*Anaerotruncus_unclassified*
*Clostridium_difficile*	*Alistipes_sp_HGB5*	*Bacteroides_faecis*
*Clostridium_glycolicum*	Bacteroides_caccae*	*Bacteroides_fragilis*
*Collinsella_intestinalis*	*Bacteroides_clarus*	*Clostridium_innocuum*
*Collinsella_unclassified*	Bacteroides_coprocola*	*Enterococcus_avium*
*Eubacterium_biforme*	*Bacteroides_finegoldii*	
*Oscillibacter_sp_KLE_1728*	*Comamonas_unclassified*	
*Paraprevotella_clara*	*Delftia_acidovorans*	
*Paraprevotella_unclassified*	*Delftia_unclassified*	
*Paraprevotella_xylaniphila*	*Ruminococcus_obeum*	
	*Subdoligranulum_sp_4_3_54A2FAA*	
HuM4	HuM5	
*Bifidobacterium_longum*	*Bacteroides_salyersiae*	
*Clostridium_leptum*	*Erysipelotrichaceae_bacterium_5_2_54FAA*	
*Coprobacillus_sp_D6*	*Roseburia_unclassified*	
*Lachnospiraceae_bacterium_1_4_56FAA*		
*Oscillibacter_sp_KLE_1745*		

*Top 5 relative abundant microbe.

Lastly, we examined how the microbial composition of the HuM mice compared relative to each other. Dendrogram clustering confirms independent and significant different clustering of each of the HuM groups (HuM1-HuM5) (Figure 4E). Interestingly, responder mice HuM2 and HuM3, cluster closely together compared to the other HuM mice (Figure 4E red and green shading). Overall, these results confirm that each of the HuM mice have different microbial communities that may influence response to immunotherapy, and that responder mice HuM2 and HuM3 cluster closely together indicating similarities in the microbiome composition and a potential mechanism to predict response to therapy in the GBM model.

## Discussion

We have recently found that transplanting gnotobiotic mice with human fecal samples results in a unique novel system of humanized microbiome mice.^20,30^ These mice are successfully colonized by human fecal microbes and maintain strain level identity and composition following breeding. We used 5 different individual healthy donors to establish 5 unique humanized microbiome mouse lines (HuM1-HuM5).^20^ Each line has a unique and significantly different microbiome composition compared to each other. We utilized healthy human control stool samples rather than patient (diseased) stool samples to first understand how human microbes affect tumor growth and response to therapy before assessing how a dysbiotic microbiome would affect these processes. We found that intracranial injection of HuM mice with GL261 cells resulted in successful formation of tumors in all 5 HuM lines, and the mice succumbed to tumor growth in untreated or vehicle control treated conditions. This is the first report to test the growth of tumors in mice that are colonized with human microbes in the GI tract. We did not see any overt differences in tumor growth rates among the 5 HuM mice when injected with GL261 cells, due to similar survival curves and median survival times as shown in Figure 2F. Others have reported that differences in the composition of the mouse gut microbiome can influence baseline tumor growth rate of melanoma flank tumors,^15^ but in the HuM1-5 mice using the GL261 model, we did not observe different growth rates. Interestingly, a recent report indicated that levels of genus *Bacteroides* change during murine glioma growth.^31^ We did not observe any significant changes in our humanized microbiome mice during tumor growth, which could have been due to differences in murine and human microbiome profiles in the mice, but we will assess this in more detail in future experiments.

Instead, we found that the various microbial communities in the HuM mice resulted in different therapeutic responses to anti-PD-1 in tumor bearing mice. We first confirmed what others have reported, using murine microbiome mice as controls, that treatment with anti-PD-1 prolongs survival in mice with GL261 intracranial tumors (Figure 3A). For the HuM mice, we found that HuM2 and HuM3 mice similarly displayed significant prolonged survival when treating with anti-PD-1 compared to isotype control. This indicated that for HuM2 and HuM3 mice, similar to MuM mice, treatment with anti-PD-1 promoted a strong anti-tumor response which resulted in decreased tumor growth rate and prolonged survival. However, when treating tumor bearing HuM1, HuM4, and HuM5 mice with anti-PD-1, a survival benefit was not observed, and the mice were resistant to the therapeutic anti-tumor immune effects of anti-PD-1 treatment. These mice are genetically identical to the HuM2 and HuM3 mice, and only differ in the composition of the gut microbiome. This indicated that the microbiome composition of HuM1, HuM4, and HuM5 led to an inability of anti-PD-1 to boost an immune response to attack the tumor. Coincidentally, this complete resistance and failure of anti-PD-1 to decrease tumor growth and prolong survival is what is observed in GBM patients. Therefore, these resistant microbiome mice are perhaps a better mouse model to study and screen therapies for GBM.

The varying efficacy to immunotherapy in the HuM mice was only observed in response to anti-PD-1, as the HuM mice were still responsive to the chemotherapy TMZ (Supplementary Figure 2). This is expected, as TMZ is an alkylating agent and directly targets proliferating GBM cells, while anti-PD-1 acts to block the immunosuppressive signals in T-cells, leading to an increased inflammatory anti-tumor response and in the end an indirect manner of tumor cell targeting and killing. Others have reported that the dosage of TMZ can dampen the efficacy of anti-PD-1 in mouse models,^32^ and that high-dose TMZ results in lymphopenia and anti-PD-1 is no longer able to boost an anti-tumor immune response. We employed a low dosage of TMZ in our studies, and to date have not tested combinatorial efforts of TMZ and anti-PD-1. Ultimately, the varying responses to anti-PD-1, and not TMZ, in the HuM mice confirm that there is a unique relationship between immune cells and microbial gut communities, which influences the competency of the immune system to further activate an anti-tumor response.

In looking more closely at the responder microbiome mice (HuM2 and HuM3), we found that *B. cellulosilyticus* was a top 5 microbe in both of these mice. There is very little evidence defining the function of *B. cellulosilyticus*, however, an extensive functional analysis has been performed on a specific strain, *B. cellulosilyticus WH2*.^33^ In this report, it was discovered that *B. cellulosilyticus WH2* was an extraordinary carbohydrate metabolizer and persevered in the guts of mice regardless of diet. However, it is not known how this particular microbe interacts with the immune cells of the gut or other potential anti-tumor effects. For HuM2 specifically, we observed an abundance of *B. caccae* that was both high in relative abundance (top 5) and exclusive to only HuM2 mice. A recent report also listed *B. caccae* as a microbe enriched in cancer patients with a positive response to anti-PD-1, indicating a potential link to our findings, but further functional validation of the specific strain of *B. caccae* found in the HuM2 mice must be performed to confirm.^34^ In our system, direct immune interactions or metabolites produced from these microbes in both HuM2 and HuM3 mice may have systemic effects in boosting an immune response, but this has yet to be confirmed.

In order to reverse the resistance from a resistant or nonresponder microbiome, others have reported that individual microbial species can assist in boosting an anti-tumor immune response.^14,15,34–36^ However, others report that it is a consortium of bacteria that exert maximal immune boosting effects.^16^ On the other hand, a recent report found that higher percentages of baseline colonic CD8^+^ T-cells did not result in fewer colitis-induced tumors.^29^ Lastly, a recent paper by D’Alessandro et al., found that administering antibiotics altered the microbiome of mice, reduced the percentage of cytotoxic cells NK (CD27^+^ CD11b^+^) leading to an increase in glioma size, with no difference in the frequency of infiltrating lymphocytes (CD45^+^ CD3^+^ T-cells).^37^ All of these reports indicate how complicated the role of the microbiome is in tumor immune interactions. In a preliminary experiment, we have found that the presence of microbes in HuM2 mice is required for the positive effect of anti-PD-1 (Supplementary Figure 3). Treating mice with a cocktail of antibiotics prevented the efficacy of anti-PD-1 in the HuM2 mice, indicating that HuM2 microbes need to be present to assist the immune response and effectiveness of anti-PD-1. Additionally, we have attempted rescue experiments and found that antibiotic depletion followed by responder microbiome fecal microbial transplant (HuM2 FMT) did not result in rescue of the resistance to anti-PD-1 in the HuM1 mice (Supplementary Figure 4). Similarly, it was reported that FMT or co-housing experiments resulted in an intermediate tumor phenotype,^29^ and that reversing microbiome profiles are robustly complicated and warrant much more investigation. Our rescue experiments are preliminary, and a more thorough characterization of therapeutic capabilities will be assessed in future studies.

Although our study represents the first report of human microbiota affecting response to immunotherapy in a mouse model of GBM, there are limitations to our findings. Unfortunately, due to our small sample size we do not have the evidence of a microbiome signature that could be of clinical translational relevance. In the HuM mice, we found that 2 of the 5 HuM lines (40%) demonstrated a positive response to anti-PD-1, which is not what was observed in the CheckMate 143 clinical trial.^5,6^ Future studies will examine whether this discrepancy is due to the human microbiota being from healthy donors (and not from GBM patients) or if this is a result of the properties of the GL261 cell line. The GL261 cell line is a highly mutated cell line, a termed “hot” tumor, that is more readily discovered and eliminated by the immune response when activated. GBM patient tumors generally exhibit a low mutational burden, a termed “cold” tumor, that is more likely undetected from immune cells even when activated by exogenous immunotherapy interventions.^4^ Furthermore, we hypothesize that the results of our study may be due to differences in immune activation from peripheral response data, but we have not assessed immune activation in the tumors of the mice. Additional intratumoral immune responses should be analyzed to determine adequate anti-tumor immune responses for the responders (HuM2 and HuM3) and examine potential exhaustion markers or lack of immune response in the resistant lines (HuM1, HuM4, and HuM5). A recent report demonstrated that fecal transplants from responder donors is safe and feasible, but correlating tumor responses to the influx of T-cells and other immune cells is still somewhat contentious.^38^ Interestingly, a peripheral immune response is being considered as a potential biomarker to assess intratumoral immune responses,^39^ but studies need to be performed to assess feasibility and validation of this approach. Lastly, the mouse microbiome is also a positive responder to anti-PD-1. This may be due to intrinsic stability of the mouse immune system and native mouse microbiota. A complicating limitation is that the mice are transplanted with human microbiota, but inevitably the mouse immune system influences the anti-tumor response. A more relevant humanized immune mouse that has been transplanted with human microbiota could perhaps shed light on this discrepancy.

In conclusion, we have found that human microbiota can influence the response to immunotherapy in a mouse model of glioma. Although the GL261 model has its limitations, it is an immune competent model and allows us to understand the role of the immune system and response to therapy in the humanized microbiome model. Understanding how microbes affect immune cells of the GI tract and systemically is important to unravel how microbiota influence anti-tumor immune responses. The question still remains of whether the “responsive” microbial communities in HuM2 and HuM3 can be therapeutically exploited, or if the “resistant” microbial communities in HuM1, HuM4, and HuM5 can be depleted and/or replaced. We have implemented a more clinically relevant model to study GBM as well as how the microbial communities can influence immune responses and anti-tumor therapies.

## Supplementary Material

vdab023_supp_Supplementary_Figure_1

vdab023_supp_Supplementary_Figure_2

vdab023_supp_Supplementary_Figure_3

vdab023_supp_Supplementary_Figure_4

vdab023_supp_Supplementary_Table_1

vdab023_supp_Supplementary_Table_2

vdab023_supp_Supplementary_Table_3

vdab023_supp_Supplementary_Material

## Notes

**Authorship Statement.** K.J.D., H.K., B.C.M., E.N.B., and C.D.M. conceived the study and wrote the manuscript; H.K., J.A.H., and D.K.C. contributed bioinformatics analyses; K.J.D., J.F.H., and B.C.M. executed all mouse experiments; M.R.C. did the NextSeq DNA sequencing; L.B.N. and E.N.B. provided reagents and reviewed the manuscript. All authors approved the final manuscript.
