# Supplementary figures and images for: Human gut microbial communities dictate efficacy of anti-PD-1 therapy in a humanized microbiome mouse model of glioma
Source: Neurooncol Adv. 2021 Feb 8;3(1):vdab023. doi: 10.1093/noajnl/vdab023 (PMC7967908; doi:10.1093/noajnl/vdab023)

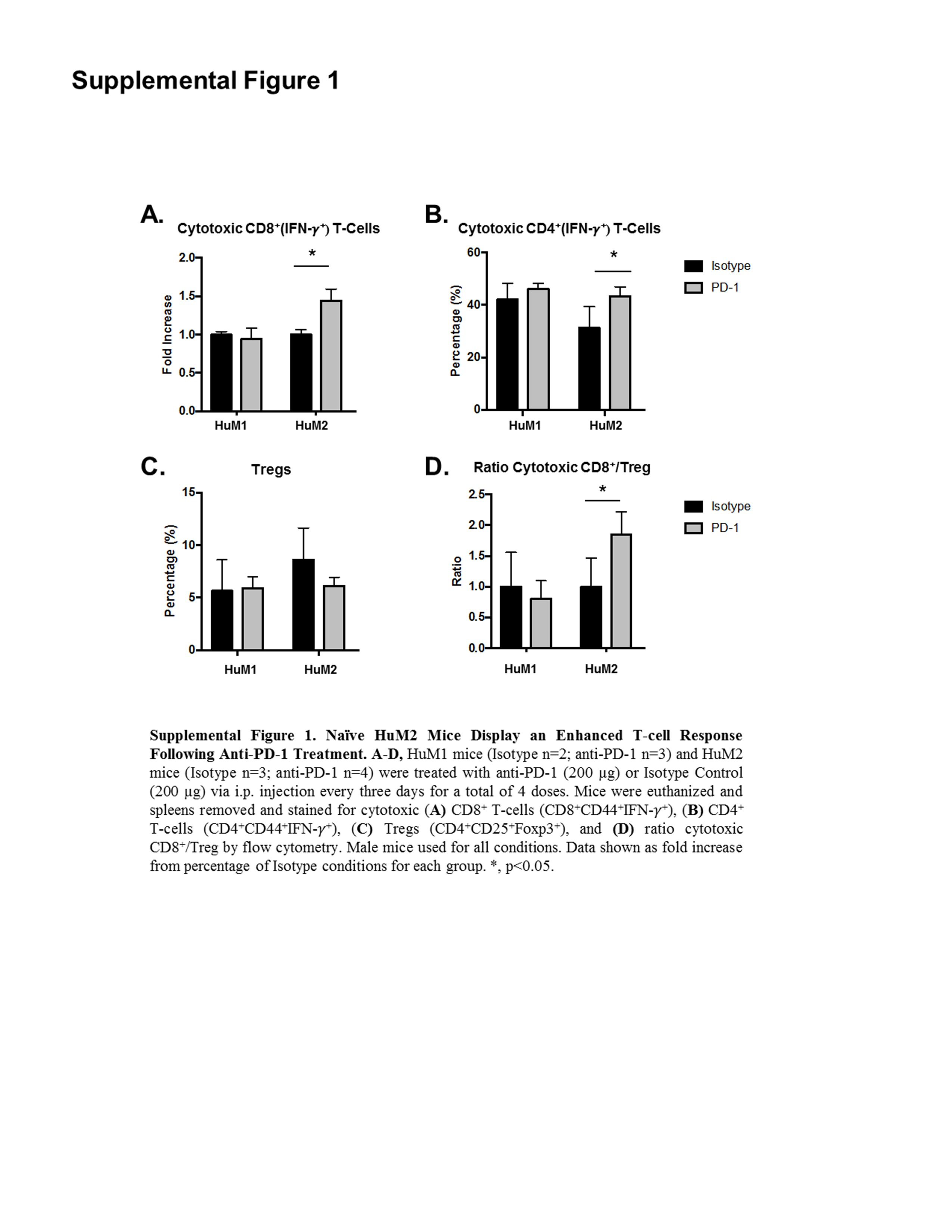

Supplement: vdab023_supp_Supplementary_Figure_1 [file vdab023_supp_supplementary_figure_1.png]

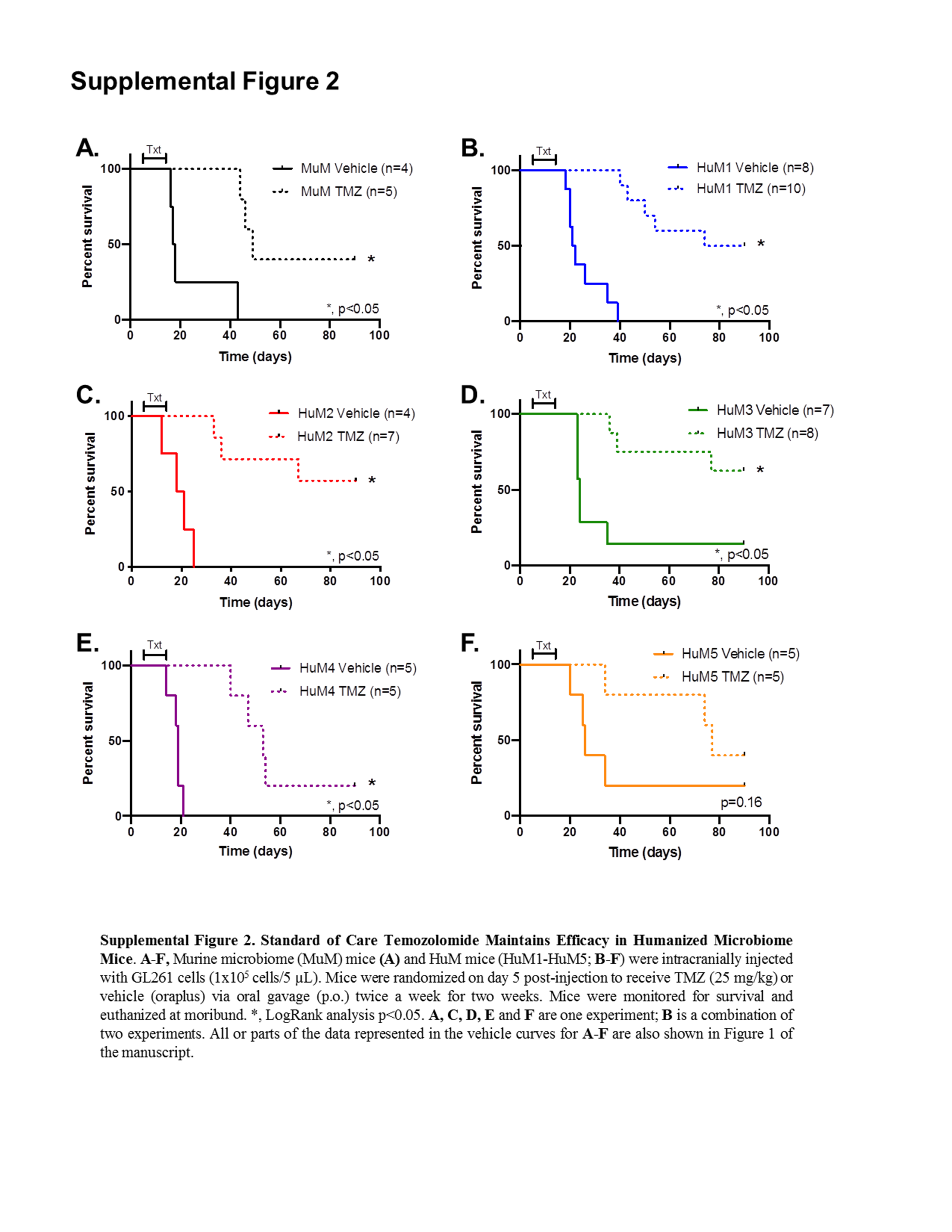

Supplement: vdab023_supp_Supplementary_Figure_2 [file vdab023_supp_supplementary_figure_2.png]

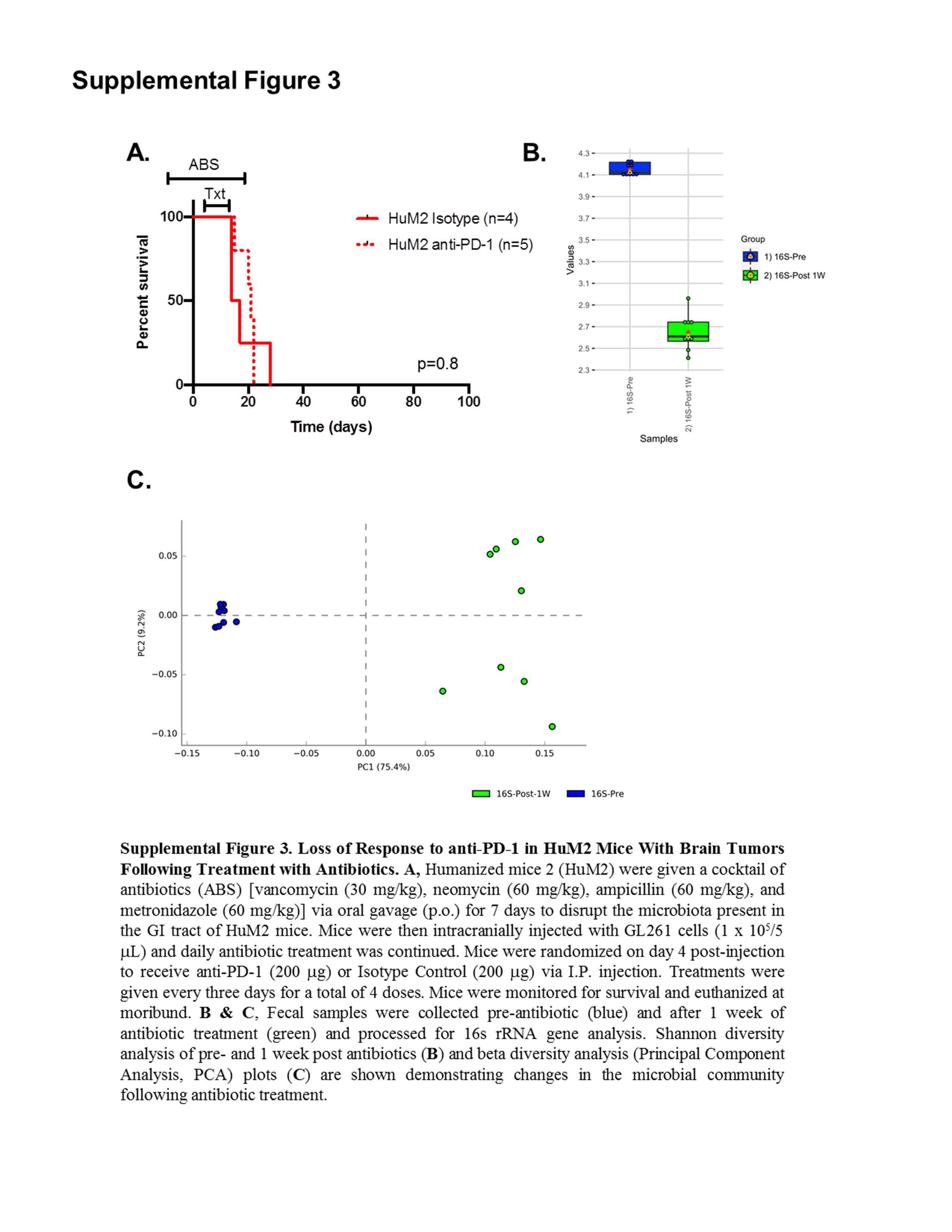

Supplement: vdab023_supp_Supplementary_Figure_3 [file vdab023_supp_supplementary_figure_3.png]

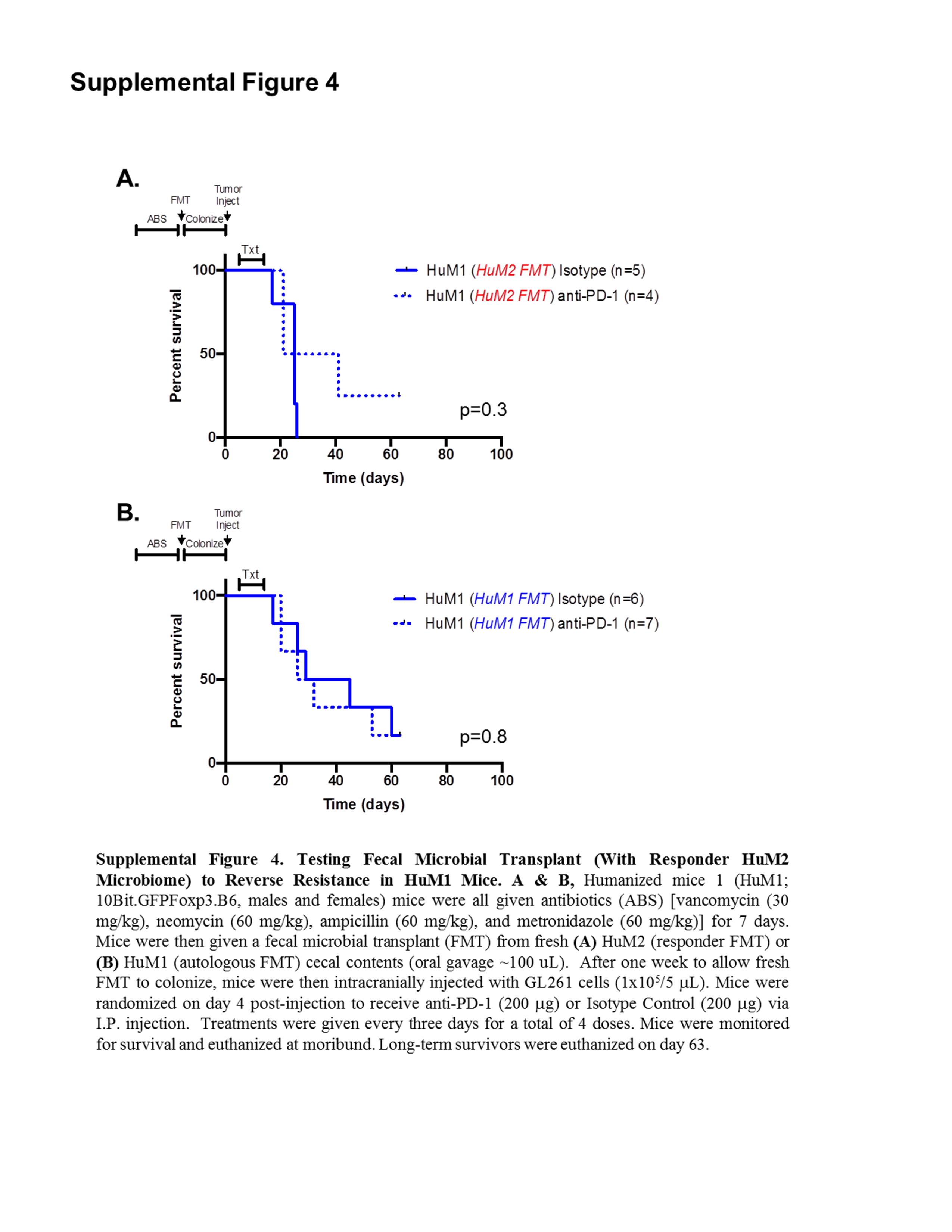

Supplement: vdab023_supp_Supplementary_Figure_4 [file vdab023_supp_supplementary_figure_4.png]
