# Supplementary material for: Human gut microbial communities dictate efficacy of anti-PD-1 therapy in a humanized microbiome mouse model of glioma
Source: Neurooncol Adv. 2021 Feb 8;3(1):vdab023. doi: 10.1093/noajnl/vdab023 (PMC7967908; doi:10.1093/noajnl/vdab023)
Supplement: vdab023_supp_Supplementary_Material [file vdab023_supp_supplementary_material.docx]

**Supplemental Methods**

*Reagents*

Antibiotics including neomycin, ampicillin, and metronidazole were purchased from Sigma, and vancomycin was purchased from Alfa Aesar. Flow cytometry antibodies for CD4 (GK1.5), CD8 (53-6.7), CD44 (IM7), Foxp3 (MF-14), CD25 (PC61) and IFN-γ (XMG1.2) were purchased from BioLegend.

*Flow Cytometry*

Spleens were isolated from the mice and passed through a 100 μm filter followed by brief ACK lysis. Cells were then incubated with antibodies to detect the following populations: CD8^+^ T-cells (CD8^+^CD44^+^IFN-γ^+^), CD4^+^ T-cells (CD4^+^CD44^+^IFN-γ^+^), and Tregs (CD4^+^CD25^+^Foxp3^+^). Samples were run on the LSRII FACS Caliber and data analyzed by FlowJo software and displayed as fold increase from frequency of the population (%) as previously described ^1^.

*Isolation of DNA from fecal Samples for 16S rRNA Analysis*

Fecal samples were collected from the HuM2 mice at pre antibiotic treatment (*n*=9) and post 1-week antibiotic treatment (*n*=9) and 16S ribosomal RNA (rRNA) gene sequencing was performed and analyzed as previously described ^2^. A Principal Component Analysis (PCA) plot was generated using Statistical Analysis of Metagenomic Profiles ^3^.

**References**

**1.** McFarland BC, Marks MP, Rowse AL, et al. Loss of SOCS3 in myeloid cells prolongs survival in a syngeneic model of glioma. *Oncotarget.* 2016; 7(15):20621-20635.

**2.** Kumar R, Eipers P, Little RB, et al. Getting started with microbiome analysis: sample acquisition to bioinformatics. *Curr Protoc Hum Genet.* 2014; 82:18.18.11-29.

**3.** Parks DH, Tyson GW, Hugenholtz P, Beiko RG. STAMP: statistical analysis of taxonomic and functional profiles. *Bioinformatics.* 2014; 30(21).
